# Surveillance of Mosquitoes (Diptera, Culicidae) in a Northern Central Region of Spain: Implications for the Medical Community

**DOI:** 10.3389/fvets.2019.00086

**Published:** 2019-04-23

**Authors:** Ignacio Ruiz-Arrondo, Barry J. McMahon, Luis M. Hernández-Triana, Paula Santibañez, Aránzazu Portillo, José Antonio Oteo

**Affiliations:** ^1^Center of Rickettsiosis and Arthropod-Borne Diseases, Hospital Universitario San Pedro-CIBIR, Logroño, Spain; ^2^UCD School of Agriculture and Food Science, University College Dublin, Dublin, Ireland; ^3^Wildlife Zoonoses and Vector-Borne Diseases Research Group, Virology Department, Animal and Plant Health Agency, Addlestone, United Kingdom

**Keywords:** surveillance, mosquito, One Health, flavivirus, molecular identification, La Rioja, Northern Spain

## Abstract

Mosquitoes are important to public and animal health due to their capacity to transmit diseases. Since the Zika virus was declared a pandemic by the WHO in 2016, and it has been recorded in different regions of Mediterranean Area (included Spain), the Government of La Rioja (Northern Spain) through the Center of Rickettsiosis and Arthropod-Borne Diseases, implemented an entomological surveillance programme of mosquitoes in La Rioja and in a close area of Navarra. This surveillance extended to some of the pathogens that they can transmit. Here we describe the framework of the initial surveillance programme for the detection of mosquitoes and associated human pathogens. We outline the benefits and the limitation of the programme to date, and explore how greater benefits can be achieved, for example using a One Health approach. Entomological surveillance has been carried out with BG-Sentinel traps, human bait technique and other methods such as collecting adults in resting places or immature stages by dipping in several wetlands. Since *Aedes albopictus*, vector of arbovirus such as Dengue, Chikungunya, and Zika, has not been detected yet in the region, the entomological programme included the surveillance of this exotic species using ovitraps in the most important cities. Morphological identification was supported using the mitochondrial cytochrome C oxidase subunit I and the internal transcribed spacer 2 genes analysis. In 2016 and 2017, more than 6,000 mosquitoes were collected. The mosquito's community included 21 species associated with six genera: *Anopheles* (*n* = 4), *Aedes* (*n* = 5), *Culex* (*n* = 6), *Culiseta* (*n* = 4), *Uranotaenia* (*n* = 1) and *Coquillettidia* (*n* = 1). Eleven species represent new records for La Rioja and Navarra regions. Several species were collected biting humans and a great proportion of the sampled mosquito population are competent vectors of several pathogens, such as West Nile virus. Sequences closely related to mosquito–only flavivirus have been detected in 0.34% of analysed pools. At the same time, the epidemiological surveillance emphasis is placed in the early detection of mosquito-borne diseases in primary health and emergency services. The surveillance programme represents a relevant and necessary assessment of the risk of pathogen transmission in a region, and it allows for the establishment of the appropriate preventive measures.

## Introduction

Mosquitoes are considered the most important arthropod vectors in the world (1, 2). Globalization in conjunction with climate change, landscape change and the capacity of mosquitoes to adapt to a changing world favour the emergence and re-emergence of numerous mosquito-borne diseases (3, 4).

Vector-borne diseases are increasing in Europe with the presence of alien and native species of mosquitoes. Thus, the invasive tiger mosquito (*Aedes albopictus*) has been involved in the transmission of Chinkungunya virus (CHIKV), and autochthonous cases of CHIKV have been reported in France and Italy from 2007 to 2017 (5, 6). *Aedes albopictus* has been also related to cases of Dengue virus (DENV) reported in France from 2010 to 2015 (7). More recently, DENV has been recorded from Spain, and again in France (8, 9). Moreover, native species such as *Culex pipiens* s.l. or *Anopheles atroparvus*, could play a prominent role in the transmission of pathogens, such as the West Nile virus (WNV) (10) or malaria, respectively (11, 12).

The emergence and resurgence of some mosquito-borne diseases has led to the implementation of mosquitoes and arboviruses surveillance programs in some European countries, in an effort to reduce the impact of these infections on public health (13). Arboviruses surveillance requires a One Health approach that integrates the health of humans, animals (livestock and wildlife), and the ecosystems to prevent disease outbreaks (14). This includes the surveillance of mosquitoes. Research on the distribution, abundance and species composition of mosquitoes in a region is vital in order to estimate the risk of incidence of vector-borne diseases (15–17).

Mosquito-borne disease surveillance programs vary among European countries, according to different environmental and socio-economic scenarios (18) and, to a greater or lesser extent, within the One Health perspective. This is, for instance, the case of West Nile disease (WND) surveillance program. WNV remains in an enzootic cycle among birds, and it does not easily adapt to urban spaces (19). Mosquitoes of the genus *Culex* are the main vectors in Europe (18), and humans and equids are accidental hosts. WNV is continuously circulating in Europe with a recent increasing trend of incidence in several European countries (20). In Spain, a country where WNV is endemic (21), a specific national surveillance plan for WNV has been carried out since 2007 in high risk areas, located mainly in southern Spain. Nevertheless, WNV screening in mosquitoes had been previously done in wetlands in western Andalucía (2001–2013) and Catalonia (2001–2009) (18). In addition to the entomological surveillance, both passive and active surveillance were carried out on birds and horses (22).

In February 2016, WHO declared Zika virus (ZIKV) infection as a public health emergency of international importance due to its rapid expansion over-wide and severe complications, including congenital microcephaly and Guillain-Barré syndrome (23). In Spain, a National Plan of preparedness and response against CHIKV, DENV, and ZIKV was then developed (23) due to the presence of *Ae. albopictus* in several regions of the country (24). At the same time, the 17 Autonomous Communities from Spain were urged to make their own plans against this mosquito threat. The latter and the lack of knowledge about the circulation of mosquitoes in La Rioja (northern central of Spain), urged La Rioja Government to implement a mosquito (and their related microorganisms) surveillance program in the region in 2016, through the Center of Rickettsiosis and Arthropod-Borne Diseases (CRETAV).

CRETAV is a reference centre for arthropod-borne diseases in Spain. A multidisciplinary team (physicians, biologists, veterinarians, entomologists, biochemists and pharmacists) works in coordination dedicated to the study of these zoonosis, focused on the One Health concept. In addition, physicians who treat patients with febrile syndromes are sensitized with the emergence and re-emergence of diseases transmitted by arthropod vectors (25–27).

Within the regional plan for surveillance of arboviruses in La Rioja, a coordinated group among the different sectors involved was formed to follow up on imported cases and adequately respond to risk situations. The key elements within this plan were: epidemiological, entomological and microbiological surveillance, entomological response, individual protection, training and information, and coordination and communication (28); thus requiring a multidisciplinary team which needs to understand the ecology of the mosquitoes. Specifically, the main measures were focused on entomological surveillance as well as epidemiological surveillance of imported cases in case of *Ae. albopictus* (and/or other competent vector) detection. The aim of this manuscript was to describe initial surveillance programme for the detection of mosquitoes and associated human pathogens. The study involved not only mapping the mosquito species distribution, but also investigating their abundance, phenology and preference for hosts. It was focused on the collection of ecological data to inform about the epidemiology of mosquito-borne diseases. We outline the benefits and the limitation of the programme to date, and explore how greater benefits can be achieved, for example using a One Health approach.

## Methodology

### Study Area

**Figure 1:**
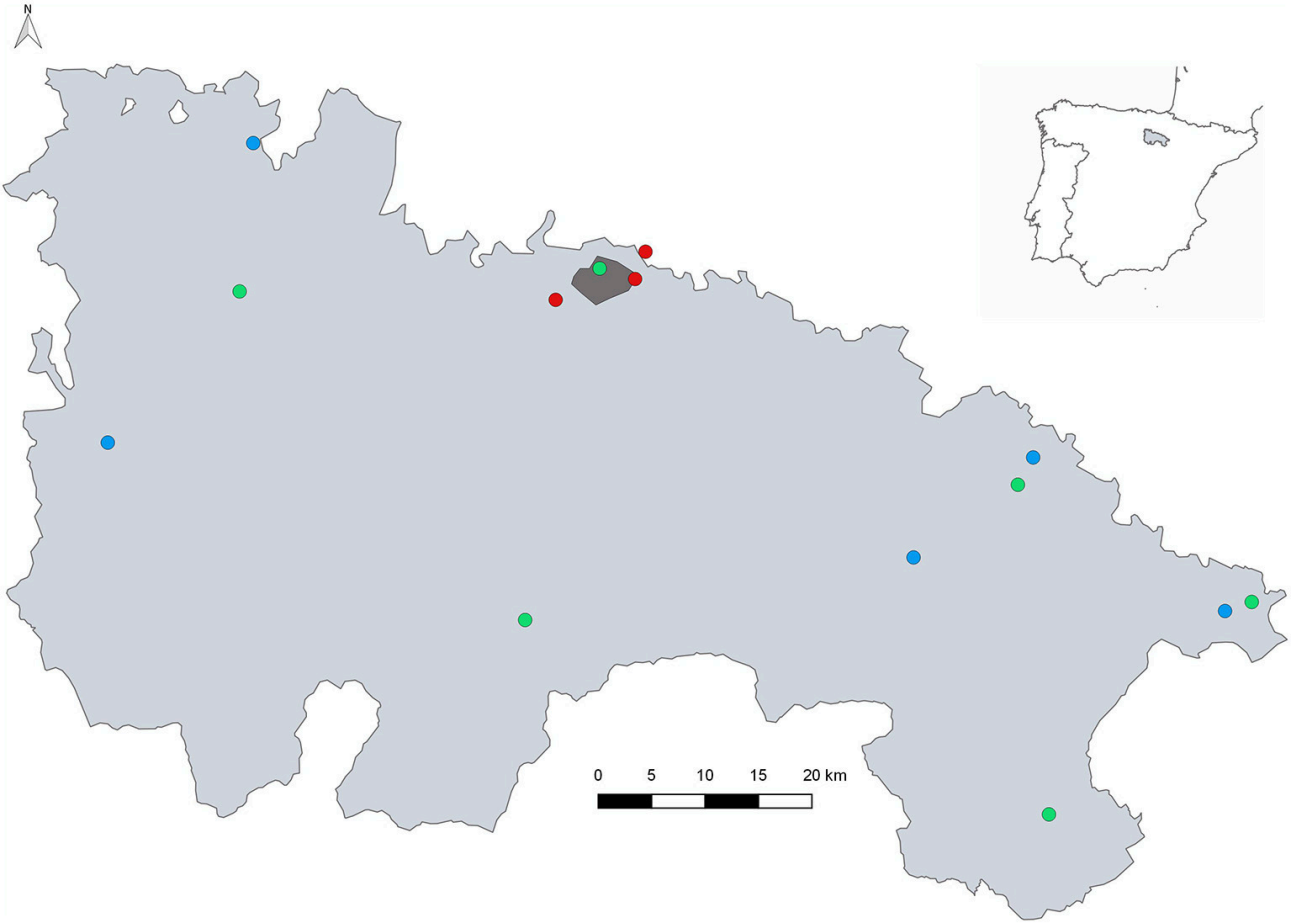
Distribution of the mosquito sampling sites. Dark grey is the urban zone of Logroño city; red circles indicate wetlands and river Iregua sampled permanently; green points are wetlands visited occasionally; and blue circles indicate the municipalities with presence of ovitraps. The map was created using QGIS 2.8.

The study area is located in the Autonomous Community of La Rioja (northern Spain) and a close area of Navarra region (Figure 1). La Rioja is a small region (5,034 km^2^ and 312,830 inhabitants) in Spain. It has different habitats with great biodiversity. Its territory expands between the plain in the North, with the Ebro river Valley, with altitudes between 300 and 400 metres above sea level (m.a.s.l.), and the mountains in the South, with the presence of several valleys with North-South direction, with maximum altitude of 2,271 m.a.s.l. (29). The climate is temperate with variations according to altitude.

The entomological surveillance encompasses areas placed in Iregua river (in Logroño) as well as La Grajera and Las Cañas wetlands (the last one in Navarra region), both located very close to Logroño (red points, Figure 1). These areas were chosen according to the presence of mosquitoes, waterfowl and migratory bird species, and because they were regularly visited by the public. This situation makes these wetlands points of specific interest for monitoring arboviruses. The entomological surveillance also included sporadic visits to other wetlands (green points, Figure 1) in order to investigate the mosquito fauna through the entire region of La Rioja. The surveillance of alien species was carried out in six municipalities (blue points and Logroño city, Figure 1). Geolocation of sampling sites are included in Table S1.

### Mosquito Collection

Entomological surveillance was carried out using different collecting techniques: Trapping devices, human landing technique and other methods, such as collecting adults in resting places or catching immature stages by dipping.

Mosquitoes were collected from July to September 2016, and from May to September 2017. A total of 16 BG-1 Sentinel™ traps (BioGents GmbH, Regensburg, Germany) baited with BG-Lure® and CO_2_ were set once every 2 weeks in wetlands.

Traps were placed at dusk and checked the following morning and, at the same time, the mosquitoes were captured by human landing technique during 10 min per trap using mouth aspirators (30). Resting adults were captured from natural and artificial hiding places and the surrounding vegetation in breeding sites by vacuuming (31) using an InsectaZooka and an AC/DC aspirators (Bioquip Products, Rancho Dominguez, CA, USA).

The entomological programme also included the surveillance of *Ae. albopictus* using ovitraps (31) in the most important cities because it was known to be present in three border regions, the Basque country, Aragón (32), and in Navarra (unpublished data). A total of 80 ovitraps locations were chosen in selected municipalities (blue points and Logroño city, Figure 1). The ovitraps were checked every 2 weeks for *Ae. albopictus* eggs from July to October 2016–2017.

### Mosquito Identification and Viruses Screening

Collected adult specimens were placed into a cooler containing dry ice and transported to the laboratory for storage at −80°C until processing. Larval specimens were preserved in 80% ethanol until mounted on slides and pupae were conserved with water from breeding place to obtain link-reared adults. Adults were separated on a chill table, according to their gender and their engorged status. Wooden sticks of the ovitraps were checked under a stereoscope in the search of eggs. If present, they were introduced into water for hatching, following the protocol of Alarcón-Elbal et al. (33). All specimens were morphologically classified using taxonomic keys (34, 35).

Molecular identification was carried out in selected adults and in unhatched eggs using PCR assays targeting the mitochondrial cytochrome C oxidase subunit I (COI) and the internal transcribed spacer 2 (ITS2) (36). A modified hotshot technique was used for DNA extraction using only leg(s) from the adult specimen (37). Eggs collected on every wooden stick were pooled, and DNA was extracted using the kit DNeasy Blood and Tissue (Qiagen, Hilden, Germany). PCR products were sequenced in both senses using the BigDye® Terminator v3.1 Cycle Sequencing Kit (Applied Biosystems, Forest City, CA, USA) at the Sequencing Unit, Center for Biomedical Research of La Rioja (CIBIR), Spain. Nucleotide sequences were compared with those deposited in GenBank using BLAST tool (www.ncbi.nlm.nih.gov/genbank), and in BOLD Systems (http://www.boldsystems.org/index.php/IDS_IdentificationRequest). Neighbour joining analyses were conducted in MEGA4. Detailed specimen records and sequence information (including trace files) are available on Barcode of Life Database (BOLD) (see http://www.boldsystems.org) and Genbank.

After identification, unfed female mosquitoes were pooled (a maximum of 50 individuals/pool) by wetland, collection date and species. The RNA was extracted from the homogenates and reverse-transcribed using RNeasy Mini Kit and Omniscript RT kit (Qiagen, Hilden, Germany), respectively, following the manufacturer's instructions and tested for flavivirus using a generic nested PCR assay (38). Japanese Encephalitis virus was used as positive control. The strains-14 was obtained through the European Virus Archive (EVAg) consortium and passed three times in Vero Cells. All procedures were carried out under sterile conditions in a Class II biosafety cabinet in a biosafety level 2 laboratory at CIBIR. PCR products were sequenced and analyzed as explained above.

## Results

### Identification of Mosquitoes

In the studied period 2016–2017, a total of 6,658 mosquitoes were collected by traps in permanently sampled wetlands. The community composition of the samples included 21 species belonging to six genera: *Anopheles* (*n* = 4), *Aedes* (*n* = 5), *Culex* (*n* = 6), *Culiseta* (*n* = 4), *Uranotaenia* (*n* = 1) and *Coquillettidia* (*n* = 1) (Table 1). Eleven species represented new records for La Rioja (*Anopheles algeriensis, Anopheles plumbeus, Aedes berlandi, Aedes cantans, Aedes vexans, Aedes detritus, Coquillettidia richiardii, Culex theileri, Culiseta litorea, Culiseta subochrea*, and *Uranotaenia unguiculata)* added to the fourteen species previously described in the region (43, 44). Five species were new records for Navarra (region nearby of La Rioja) (*An. algeriensis, An. plumbeus, Ae. detritus, Cs. litorea* and *Cs. subochrea*) along with the fourteen species previously reported (45, 46). During the surveillance in 2016, eggs that morphologically seemed compatible with those from *Ae. albopictus* were detected, although the identification could not be confirmed by molecular methods. To date, *Ae. albopictus* has not been detected in La Rioja or in the studied area of Navarra.

**Table 1:** Mosquito species captured with different collection methods and vector competence for humans (34, 35, 39–42).

**Species**	**Collection method**				**Vector competence (confirmed in laboratory)**
	**T**	**HB**	**R**	**D**	
*Anopheles algeriensis* Theobald, 1903	x		x		*Plasmodium* sp.
*Anopheles atroparvus* Van Thiel, 1927	x		x	x	*Plasmodium* sp. WNV
*Anopheles claviger* s.l. (Meigen, 1804)	x		x		*Plasmodium* sp.
*Anopheles plumbeus* Stephens, 1828	x	x			*Plasmodium* sp. WNV
*Aedes berlandi* Seguy, 1921	x				-
*Aedes cantans* (Meigen, 1818)		x			Tahyna virus WNV
*Aedes caspius* (Pallas, 1771)	x	x	x		Tahyna virus WNV
*Aedes detritus* (Haliday, 1833)	x	x			JE virus WNV
*Aedes vexans* (Meigen, 1830)	x	x			EEE virus RVF virus Tahyna virus WNV
*Culex hortensis* Ficalbi, 1889	x			x	-
*Culex impudicus* Ficalbi, 1890	x		x	x	-
*Culex mimeticus* Noè, 1899	x				WNV
*Culex modestus* Ficalbi, 1889	x			x	Lednice virus Tahyna virus WNV
*Culex pipiens* s.l. Linnaeus, 1758	x		x	x	Sindbis virus Usutu virus JE virus SLE virus RVF virus WNV
*Culex theileri* Theobald, 1903	x		x	x	Sindbis virus RVF virus WNV
*Culiseta annulata* (Schrank, 1776)	x		x	x	WNV
*Culiseta longiareolata* (Macquart, 1838)	x		x	x	-
*Culiseta litorea* (Theobald, 1901)	x			x	-
*Culiseta subochrea* (Edwards, 1921)	x		x	x	-
*Coquillettidia richiardii* (Ficalbi, 1889)	x	x	x		WNV
*Uranotaenia unguiculata* Edwards, 1913	x		x	x	-

*T, BG-1 Sentinel™ trap; HB, Human-bait; R, Rest; D, Dipping; EEE, Easter equine encephalitis; JE, Japanese encephalitis; SLE, Sant Louis encephalitis; RVF, Rift Valley fever*.

**Figure 2:**
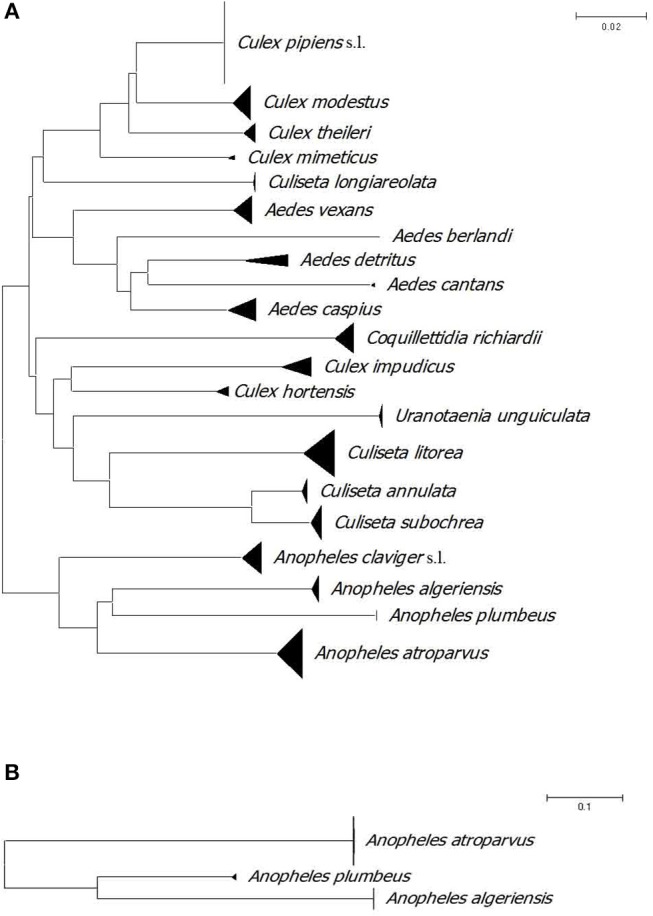
**(A)** Neighbour joining tree of COI DNA barcodes (658 bp) for mosquito species collected in La Rioja, Spain. **(B)** Neighbour joining tree of ITS2 sequences (475 bp) for *Anopheles* species collected in La Rioja, Spain. A divergence of >2% might be indicative of separate operational taxonomic units.

Table 1 shows the capture methods for each species with six species that were collected biting humans: *An. plumbeus, Ae. cantans, Aedes caspius, Ae. detritus, Ae. vexans, and Cq. richiardii*. All identified species have been molecularly confirmed. In total, we obtained 262 full length 658 bp barcodes for COI and 47 barcodes for ITS2. The neighbour joining (NJ) trees show that all specimens belonging to the same species based upon morphological characters grouped together in the tree (Figure 2). *Anopheles claviger* s.l. was not included in the ITS2 NJ tree because of failure of DNA amplification. In addition, for those samples that showed PCR products, the obtained sequences were too short to be included in the dataset.

Regarding blood-fed, 341 female specimens were collected. Forty per cent of the samples were caught in the BG-1 Sentinel™ traps, and the remaining 60% in resting places.

The composition and the abundance of the species varied depending on the wetland (see Table 2). In La Grajera wetland, *Cq. richiardii* (43.7%), *Cx. pipiens* s.l. (16.9%), *An. algeriensis* (15.9%) and *An. claviger* s.l. (11.5%) were the most abundant species, whereas in Las Cañas wetland, *Ae. caspius* (47%) and *Cx. pipiens* s.l. (23%) were the main collected species. In Iregua river, *Cx. pipiens* s.l. (64.4%) and *Cx. modestus* (12.5%) were the most common species. In occasionally sampled wetlands, all but *Aedes cantans* species were the same as those found in permanently sampled sites. Phenology and ecological data have been obtained and those from 2017 in La Grajera wetland have been already published (30).

**Table 2:** Number of mosquitoes (per species and sex), percentage of relative abundance and distribution in the permanently sampled wetlands during 2016–2017.

**Species**	***n***	**%**	**F**	**M**	**Iregua**		**La Grajera**		**Las Cañas**	
*An. algeriensis*	617	9.3%	613	4	0	0.0%	587	15.9%	30	1.2%
*An. claviger* s.l.	441	6.6%	440	1	9	2.1%	427	11.5%	5	0.2%
*An. maculipennis* s.l.	36	0.5%	19	17	2	0.5%	6	0.2%	28	1.1%
*An. plumbeus*	5	0.1%	4	1	4	0.9%	0	0.0%	1	0.0%
*An*. spp.	33	0.5%	33	-	0	0.0%	32	0.9%	1	0.0%
*Ae. berlandi*	1	0.0%	1	0	0	0.0%	1	0.0%	0	0.0%
*Ae. caspius*	1,245	18.7%	1,245	0	6	1.4%	53	1.4%	1,186	47.0%
*Ae. detritus*	10	0.2%	10	0	0	0.0%	2	0.1%	8	0.3%
*Ae. vexans*	64	1.0%	44	20	0	0.0%	60	1.6%	4	0.2%
*Ae*. spp.	40	0.6%	40	-	0	0.0%	0	0.0%	40	1.6%
*Cq. richiardii*	1,857	27.9%	1,814	43	8	1.9%	1,616	43.7%	233	9.2%
*Cx. pipiens* s.l.	1,504	22.6%	1,350	154	299	69.4%	625	16.9%	580	23.0%
*Cx. modestus*	348	5.2%	348	0	54	12.5%	77	2.1%	217	8.6%
*Cx. mimeticus*	4	0.1%	1	3	3	0.7%	1	0.0%	0	0.0%
*Cx. theileri*	160	2.4%	158	2	2	0.5%	77	2.1%	81	3.2%
*Cx. impudicus*	4	0.0%	4	0	0	0.0%	4	0.2%	0	0.0%
*Cx*. spp.	96	1.4%	96	-	21	4.9%	33	0.9%	42	1.7%
*Cs. annulata*	40	0.6%	40	0	5	1.2%	27	0.7%	8	0.3%
*Cs. longiareolata*	29	0.4%	22	7	11	2.6%	18	0.5%	0	0.0%
*Cs. litorea*	30	0.5%	27	3	4	0.9%	17	0.5%	9	0.3%
*Cs. subochrea*	88	1.3%	88	0	3	0.7%	37	1.0%	48	1.9%
*Cs*. spp.	1	0.0%	1	-	0	0.0%	1	0.0%	0	0.0%
*Ur. unguiculata*	5	0.1%	4	1	0	0.0%	1	0.0%	4	0.2%
Total	6,658		6,369	256	431		3,702		2,525	

*Aedes cantans and Cx. hortensis are not included in the table because they were identified during occasional samplings in 2018. F, Female; M, Male*.

### Screening for Flaviviruses

Up to date, four pools (0.34%), three from La Grajera wetland and one from Las Cañas wetland, from *Ae. vexans* captured in 2016 and 2017, have yielded positive results for flavivirus PCR screening (Table 3). They showed maximum identity (97-99%) with the sequences of *Aedes vexans* flavivirus (AeveFV) group deposited in GenBank (GQ476996- GQ476998, GQ477000 and JN802280).

**Table 3:** Pools of unfed female specimens from each mosquito species tested up to the moment for this project.

**Mosquito species**	**Number of tested mosquitoes**	**Number of tested pools**	**PCR results for flavivirus screening**
*Anopheles algeriensis*	307	15	-
*Anopheles claviger* s.l.	36	3	-
*Anopheles maculipennis* s.l.	14	3	-
*Anopheles plumbeus*	-	-	
*Aedes berlandi*	-	-	
*Aedes cantans*	1	1	-
*Aedes caspius*	135	9	-
*Aedes detritus*	3	1	-
*Aedes vexans*	18	4	4
*Culex hortensis*	-	-	
*Culex impudicus*	-	-	
*Culex mimeticus*	1	1	-
*Culex modestus*	106	8	-
*Culex pipiens* s.l.	573	24	-
*Culex theileri*	94	9	-
*Culex* spp.	11	2	-
*Culiseta annulata*	3	1	-
*Culiseta longiareolata*	9	2	-
*Culiseta litorea*	6	5	-
*Culiseta subochrea*	26	7	-
*Coquillettidia richiardii*	523	21	-
*Uranotaenia unguiculata*	-	-	
Total	1,866	116	4

## Discussion

This is the first study performed in northern central Spain focused on the investigation of the mosquito species and their potential infections with flaviviruses. In Spain, mosquito screening for arboviruses had been previously performed in other regions, like Catalonia and Western Andalusia (47–49). The study has allowed us to identify numerous mosquito species with vector capacity as well as providing an insight into the ecology of these vectors. Sequences closely related to mosquito–only flavivirus have been detected in the analysed samples. The surveillance of mosquito's circulation is very important for the clinical practice since travellers affected by DENV, CHIKV and ZIKV have been diagnosed in the country, and because the first autochthonous cases of Dengue have been reported in Spain as well as in neighbouring countries (8).

Since *Ae. albopictus* has not been detected in the region, the risk of autochtonous transmission of arbovirus like DENV, CHIKV, and ZIKV remains very low. This fact means that the next level of action within the regional plan for surveillance of arboviruses in La Rioja should not be extended and, among other measures, epidemiological surveillance and control of the vector have not been necessary in the case of imported cases of arboviruses.

In this study, six mosquito species were found biting humans. Some species, like *Ae. caspius* or *Cq. richiardii*, are very abundant in the studied area and could act as bridge vectors for pathogens such as WNV, a virus that is endemic in Europe (41). *Anopheles plumbeus* is the only human-biting anopheline species out of four anopheline species identified herein. This finding suggests that despite its scarcity in the studied area, this species could be the responsible one for the case of autochthonous malaria by *Plasmodium vivax* that occurred in 2014 in Viana (Navarra), just a few kilometres away from Logroño (50). *Anopheles plumbeus* is considered a secondary vector of malaria in Europe, but it was implicated as potentially responsible for the transmission of *Plasmodium falciparum* in Germany (51). Nevertheless, *An. atroparvus* should not be ruled out as the causative agent. This species, which is more frequent in our area (Table 2), is the recognized main vector of malaria in Europe and it was involved in the transmission of the autochthonous malaria case occurred in Spain in 2010 (11). In addition, *An. atroparvus* has a wider distribution and activity range in La Rioja since their breeding sites are not restricted to water-filled holes of trees, and it can breed in a large collection of water bodies such as lagoons, irrigation channels, etc. Several studies have demonstrated that *P. vivax* is well-adapted to European populations of both *Anopheles* species (52–54). The establishment of the mosquito surveillance programme in La Rioja has contributed to increase the knowledge about the diversity, distribution abundance and ecology of species that are present in the region. These factors may determine the incidence of vector-borne pathogens in vertebrate hosts (55). In the “Big-Data era,” the generation of data about the geographic distribution will be useful to recognize possible hotspots for an outbreak and then to start the implementation of preventive measures.

In order to expand the diversity of identified species, different methodologies for mosquito collection were used. Adult trapping is most commonly used to capture flying mosquitoes (31). There are species (e.g. *An. atroparvus, Cx. impudicus*, and *Ur. unguiculata*) that have barely been detected using the BG-1 Sentinel™ traps. This could explain their scarcity in the area. However, the capture of resting mosquitoes has shown that these species mentioned above are more abundant in the sampled wetlands than previously thought. In addition, this technique made possible the capture of numerous engorged females (31).

The molecular identification of mosquitoes proved to be a useful tool to support the morphological identification. Correct identification of mosquito vectors is critical to define pathogen transmission pathways and it is the first step for preventing arboviruses transmission. The use of two genetic markers increased our taxonomic resolution (36). This molecular approach, not only helped us to identify damaged specimens and to distinguish species within a complex, but also allowed us to detect taxonomic errors based on morphological identification alone (36). Nevertheless, we could not obtain the complete fragment ITS2 gene (species-specific for *Anopheles*) studied for *An. claviger* sibling species. Kampen et al. (56) previously described also lower ITS2 region lengths for the *An. claviger* s.l. members than for other species of *Anopheles*. Both species of the complex, *Anopheles claviger* s.s. and *Anopheles petragnani* had been previously reported for several breeding sites in La Rioja region; although their morphological identification was based on preimaginal stages (44). In our study, adult specimens from these two sibling species were morphologically indistinguishable. A deeper study on the molecular identification of this anopheline mosquito complex is required. Molecular identification of all the captured individuals is unsustainable from a cost-effective point of view. However, this tool is highly recommended in groups of species very similar each other that are difficult to identify by classical morphologic and morphometric parameters, such as *Cx. impudicus-Cx. hortensis-Cx. territans*, and *Cs. litorea-Cs. morsitans-Cs. fumipennis* in our case. This approach has also been developed in other vector surveillance programs in a number of European countries [e.g., (16, 57, 58)].

The screening for flaviviruses allowed the detection of four genomic sequences closely related to mosquito-only flavivirus group. The sequences showed the highest similarity to flavivirus amplicons of AeveFV group detected in *Ae. vexans* in Italy and Czech Republic (59, 60). This is the first report of this AeveFV group in mosquitoes in Spain, although it has been detected previously by another group (Ana Vázquez, personal communication). However, the length of the obtained amplicons did not allow complete phylogenetic characterization. All *Ae. vexans* pools tested from 2016 to 2017 were positive for RNA flavivirus detection, suggesting active circulation of this flavivirus in this species. Other mosquito-only flaviviruses had been previously detected in Spain in several mosquito species including *Ae. vexans* (47, 61, 62). Further analyses of these results are necessary to characterize this flavivirus. The low number of specimens screened for flaviviruses (screening is on-going) does not allow to obtain further conclusions, specially taking into account the low prevalence of pathogenic WNV and Usutu virus (USUV) found in *Cx. perexiguus* (1.5%) and *Cx. pipiens* s.l. (0.05%) in Spain (18).

The number of mosquitoes captured in this project is lower compared to other regions from Spain where mosquitoes have been monitored (18). To date, no cases of WNV and USUV have been reported in humans, equids or in birds in northern central Spain, suggesting that there is no circulation of these viruses or, at least, their prevalence is low. In addition, this study adds new species for flaviviruses screening in Spain (e.g., *Ae. cantans, Cs. litorea*, and *Cx. mimeticus*) and significantly increases the number of specimens of certain species such as *An. claviger* s.l. and *Cq. richiardii*.

A surveillance of emerging vector-borne infections integrating the animal-human-vector approach is costly to maintain on a long-term basis (49, 63, 64). Therefore, surveillance have to adapt to the existing reality and cost-effective use of resources at the national and regional levels (14, 18, 65). The entomological surveillance started in La Rioja represents a good approach to the diagnosis of the situation of possible arboviruses in the region and may provide insights into the change in the force of infection (66) before there is an ecological alteration that may impact on human or animal health. To implement a One Health approach, it would be interesting to complete this surveillance with the screening for flaviviruses in wild birds or other potential sentinel animal species, from wetlands of interest and to include serological testing of sentinel horses. The coordinating efforts from biologists and veterinarians (18, 65, 67) would be an added value to the ongoing efforts to be aware of medical records and reports of imported mosquito-borne arbovirus human cases in this area. This approach would enable the ecological data to be operationalised to inform human, animal and ecosystem health.

## Data Availability

Detailed mosquito records and sequence information and can be found on BOLD within the Working Group 1.4 Initiative Human Pathogens and Zoonoses, in the project entitled “Surveillance of Mosquitoes in La Rioja, Spain [LRMQS, MLQSR, MQSLR].” The Digital Object Identifier (DOI) for the BOLD project is: dx.doi.org/10.5883/DS-MQSVLR. Generated sequences were also deposited in GenBank database under accession numbers MK402666 - MK402927 for COI and MK412721 - MK412767 for ITS2. Flavivirus sequence identified was also deposited in GenBank database under under accession number MK501751.

## Author Contributions

IR-A and JO designed the study. IR-A conducted the surveys and the morphological and molecular identification. IR-A and LH-T performed the molecular analysis. IR-A and PS conducted the screening for flavivirus. IR-A, BM, AP, and JO outlined the structure of the manuscript. IR-A compiled the main information and wrote the first draft of the manuscript. All authors reviewed and approved the final manuscript.

### Conflict of Interest Statement

The authors declare that the research was conducted in the absence of any commercial or financial relationships that could be construed as a potential conflict of interest.

## Abbreviations

CHIKV: Chinkungunya virus
DENV: Dengue virus
USUV: Usutu virus
WNV: West Nile virus
ZIKV: Zika virus.
