# Supplementary material for: Surveillance of Mosquitoes (Diptera, Culicidae) in a Northern Central Region of Spain: Implications for the Medical Community
Source: Front Vet Sci. 2019 Apr 23;6:86. doi: 10.3389/fvets.2019.00086 (PMC6489427; doi:10.3389/fvets.2019.00086)
Supplement: Supplementary file 1 [file Table_1.docx]

| **ID** | **Locality** | **State/Province** | **Exact Site** | **Sampling** | **Latitude** | **Longitude** | **Elevation (m)** |
| --- | --- | --- | --- | --- | --- | --- | --- |
| 1 | Ajamil | La Rioja | Rabanera wetland | Occasionally | 42º 11.381' N | 2º 28.483' W | 1073 |
| 2 | Alfaro | La Rioja | Ebro river | Occasionally | 42º 11.798 N | 1º 43.352 W | 271 |
| 3 | Alfaro | La Rioja | Urban zone | Ovitraps | 42º 10.632' N | 1º 45.015' W | 310 |
| 4 | Arnedo | La Rioja | Urban zone | Ovitraps | 42º 13.541' N | 2º 6.065' W | 524 |
| 5 | Calahorra | La Rioja | La Degollada wetland | Occasionally | 42º 16.356' N | 1º 57.373' W | 333 |
| 6 | Calahorra | La Rioja | Urban zone | Ovitraps | 42º 18,416' N | 1º 58,038 W | 352 |
| 7 | Cervera del rio Alhama | La Rioja | Linares river | Occasionally | 42º 2.951' N | 1º 55.407' W | 549 |
| 8 | Ezcaray | La Rioja | Urban zone | Ovitraps | 42º 19.457' N | 3º 0.694' W | 813 |
| 9 | Haro | La Rioja | Urban zone | Ovitraps | 42º 34,289' N | 2º 51,317 W | 478 |
| 10 | Logroño | La Rioja | Ebro river | Occasionally | 42º 28.176N | 2º 26.919 W | 368 |
| 11 | Logroño | La Rioja | Urban zone | Ovitraps | 42º 27.147' N | 2º 28.005' W | 403 |
| 12 | Logroño | La Rioja | La Grajera wetland | Permanently | 42º 26.404' N | 2º 30.577' W | 445 |
| 13 | Logroño | La Rioja | Iregua river | Permanently | 42º 26.174' N | 2º 25,648' W | 396 |
| 14 | Viana | Navarra | Hervias wetland | Occasionally | 42º 27.223 N | 2º 51.911 W | 639 |
| 15 | Viana | Navarra | Las Cañas wetland | Permanently | 42º 29.063'N | 2º 24.426' W | 376 |

**Table S1**. Geolocation of the sampling sites. Localities where surveillance of alien species using ovitraps is only referenced the locality.

| ID | **Locality** | **State/Province** | **Exact Site** | **Sampling** | **Latitude** | **Longitude** | **Elevation (m)** |
| --- | --- | --- | --- | --- | --- | --- | --- |
| 1 | Ajamil | La Rioja | Rabanera wetland | Occasionally | 42º 11.381' N | 2º 28.483' W | 1073 |
| 2 | Alfaro | La Rioja | Ebro river | Occasionally | 42º 11.798 N | 1º 43.352 W | 271 |
| 3 | Alfaro | La Rioja |  | Ovitraps | 42º 10.632' N | 1º 45.015' W | 310 |
| 4 | Arnedo | La Rioja |  | Ovitraps | 42º 13.541' N | 2º 6.065' W | 524 |
| 5 | Calahorra | La Rioja | La Degollada wetland | Occasionally | 42º 16.356' N | 1º 57.373' W | 333 |
| 6 | Calahorra | La Rioja |  | Ovitraps | 42º 18,416' N | 1º 58,038 W | 352 |
| 7 | Cervera del rio Alhama | La Rioja | Linares river | Occasionally | 42º 2.951' N | 1º 55.407' W | 549 |
| 8 | Ezcaray | La Rioja |  | Ovitraps | 42º 19.457' N | 3º 0.694' W | 813 |
| 9 | Haro | La Rioja |  | Ovitraps | 42º 34,289' N | 2º 51,317 W | 478 |
| 10 | Logroño | La Rioja | Ebro river | Occasionally | 42º 28.176N | 2º 26.919 W | 368 |
| 11 | Logroño | La Rioja |  | Ovitraps | 42º 27.147' N | 2º 28.005' W | 403 |
| 12 | Logroño | La Rioja | La Grajera wetland | Permanently | 42º 26.404' N | 2º 30.577' W | 445 |
| 13 | Logroño | La Rioja | Iregua river | Permanently | 42º 26.174' N | 2º 25,648' W | 396 |
| 14 | Viana | Navarra | Hervias wetland | Occasionally | 42º 27.223 N | 2º 51.911 W | 639 |
| 15 | Viana | Navarra | Las Cañas wetland | Permanently | 42º 29.063'N | 2º 24.426' W | 376 |
